# Adherence to the planetary health diet is associated with slower cognitive decline: a prospective cohort analysis of Chinese older adults

**DOI:** 10.1186/s12966-025-01759-y

**Published:** 2025-05-16

**Authors:** Lulu Tang, Xiaoli Yu, Changcui Qiu, Yu Lu, Yunlan Wang, Fei Liu, Xiaoping Zhu

**Affiliations:** 1 Department of Nursing, Shanghai Tenth People’s Hospital, Tongji University School of Medicine, Shanghai, China; 2 Tongji University School of Medicine, Shanghai, China

**Keywords:** Planetary health diet, Cognition, Physical activity, Older adults

## Abstract

**Background:**

The EAT-Lancet commission has proposed a planetary health reference diet (PHD) aiming to improve human health and global environmental sustainability. Emerging evidence suggests that high-quality diet is a modified risk factor cognitive decline. However, population-based evidence in relation to the association between this diet and cognitive decline is scarce.

**Methods:**

This prospective cohort study examined data from the China Health and Nutrition Survey (CHNS) during 1997–2011.We included 3404 adults aged 60 years and older with normal cognition at baseline. Dietary intake was assessed using 3-day 24 h dietary recalls combined with weighing methods and cognitive function was assessed using repeated measures of the Telephone Interview for Cognitive Status-modified (TICS-m). The Planetary Health Diet Index (PHDI) was based on 14 food groups and a total score from 0 to 140. Higher scores indicated greater adherence to the PHDI. We used linear mixed model with random intercepts and slope to evaluate the association between PHDI score and cognitive decline adjusting for demographic, health, and lifestyle confounders.

**Results:**

During a median follow-up of 12 years, higher adherence to the PHDI was associated with a slower decline in memory and global cognition. Participants in the highest quintile of PHDI adherence had significantly slower memory decline (B = 0.025, 95%CI:0.000-0.049, *P* for trend = 0.019) and global cognitive decline (B = 0.020, 95%CI:0.004–0.037, *P* for trend = 0.029) compared to those in the lowest quintile. Stratified analyses revealed that physical activity modified these associations (*P* < 0.05). Among participants engaging in vigorous physical activity, those in the highest PHDI quintile exhibited an attenuated annual memory decline (B = 0.070, 95%CI:0.010–0.130, *P* for trend = 0.013) and global cognitive decline (B = 0.045, 95%CI:0.003–0.086, *P* for trend = 0.037) compared to those in the lowest quintile.

**Conclusion:**

Higher adherence to the PHD was associated with slower cognitive decline in older Chinese adults. Physical activity, particularly vigorous physical activity, may enhance the cognitive benefits of this dietary pattern. These findings highlight the potential dual benefits of sustainable dietary patterns for both environmental and cognitive health.

**Supplementary Information:**

The online version contains supplementary material available at 10.1186/s12966-025-01759-y.

## Introduction

Modern food systems represent a major driver of global climate change, environmental degradation, contributing approximately 30% of global greenhouse gas emissions [1], 40% of land use, and 70% of available freshwater use [2, 3]. The interconnected crises of planetary health and human health are increasingly evident. Low-quality diets have led to an increase in chronic metabolic disease prevalence (e.g. type 2 diabetes, cardiovascular diseases, obesity) and global death [4, 5, 6, 7]. Simultaneously, the global prevalence of unhealthy dietary patterns, particularly Western-style diets characterized by excessive consumption of sugars, salt, processed foods and animal products, has exacerbated environmental degradation through substantial water misuse and biodiversity loss [8, 9]. Therefore, transitioning toward dietary patterns that simultaneously support human health and environmental sustainability has become imperative.

Cognitive function refers to the neurobiological processes of receiving and transforming information, encompassing visual and auditory processing, memory, and fluid reasoning. Accelerated global population aging has established age-related cognitive decline as a primary driver of dementia spectrum disorders (DSDs), including Alzheimer’s disease (AD), Parkinsonism dementias, vascular dementia, and mild cognitive impairment (MCI) [10]. DSDs currently affect over 55 million adults worldwide, with more than 60% residing in low-and-middle-income countries (LMICs) [11]. As the world’s most populous LMIC, China exhibits strikingly high prevalence rates of DSDs. Epidemiological surveillance demonstrates that 15.5% of Chinese adults aged ≥ 60 years meet diagnostic criteria for MCI, while dementia and AD prevalence rates reach 6.0% and 3.9%, respectively [12]. These figures underscore the critical need for effective preventive strategies. Given the essential role of nutrients and dietary patterns in neuroendocrine regulation, dietary interventions have emerged as promising strategies for delaying the progression of cognitive decline in public health frameworks [13, 14, 15]. The significant associations of dietary patterns (e.g. Mediterranean diet, anti-inflammatory diets, plant-based diets, and Mediterranean-DASH Intervention for Neurodegenerative Delay (MIND) diet) with reduced cognitive decline in older persons have also been previously reported [16, 17, 18]. However, these dietary patterns mainly focused on human health outcomes while incorporating diverse food products, leading to substantial variations in dietary quality indices.

In 2019, the EAT-Lancet Commission proposed a planetary health reference diet (PHD) to establish global scientific targets for healthy diets and sustainable food systems [19]. Synthesizing evidence from food consumption patterns and disease prevention research, the PHD provides evidence-based recommendations that surpass conventional national dietary guidelines in addressing global population growth. Characterized by plant-based dominance, the PHD specifies limitations on animal-derived products (particularly meat and dairy) while emphasizing increased consumption of vegetables, fruits, whole grains, and nuts. Existing observational studies have demonstrated that adherence to the PHD is associated with lower risk of multiple diseases and mortality using large sample data [20, 21, 22, 23]. Diet has attracted much attention as a modifiable factor on cognitive aging [24]. A study in Brasil of 11,737 adults showed a protective effect of PHD on cognitive decline [25]. A Singapore national cohort of 16,736 participants found that adherence to PHD was associated with reduced risk of poor cognitive function [26]. Although prospective cohort studies suggest potential protective effects [27, 28], these investigations have focused on specific cultural dietary contexts or high-income countries, lacking representation from LMICs. China is facing dual challenges of substantial environmental pressures and rising diet-related cognitive decline risks, highlighting the urgency of implementing sustainable dietary strategies. To date, no large-scale studies have evaluated the relationship of the PHD with cognition benefits within Chinese populations, which limited the capacity of identifying important associations between PHD and cognition in the general population.

Advanced age remains the most robust biological risk factor for dementia, as neurodegenerative pathology exhibits accelerated accumulation beyond 60 years [29]. China has 249.49 million people aged 60 years or older, representing 17.9% of the total population of 1.40 billion people [30], suggesting a high prevalence of DSDs. While preliminary evidence from a UK cross-sectional study (*n* = 18–35 years) suggests potential neuropsychological benefits of the PHD [31]. However, that study examined relatively young adults, who were less likely to have poor cognitive function compared with older adults. Consequently, the protective effects of adherence to PHD against age-related cognitive decline in older adults remains insufficiently characterized.

To operationalize adherence to the PHD, we employed the Planetary Health Diet Index (PHDI) developed by Cacau et al. that includes 14 food groups range from 0 to 140 [32 The PHDI reflects the degree of adherence to the PHD. In our study, we aimed to investigate the prospective associations between adherence to the cumulative average PHDI and cognitive decline in a population-based cohort study in China over 7 years of follow-up. We hypothesized that higher adherence to the PHDI would be related to slower cognitive decline among Chinese adults aged 60 years or older.

## Methods

### Study design and participants

The China Health and Nutrition Survey (CHNS) is a long-term prospective cohort study among Chinese recruited from general population based on multiple cross-sectional surveys conducted during 1989-2015 [33]. All participants provided written informed consent covering both baseline assessment and follow-up surveys. The study protocol received ethical approval from research ethics committees of the University of North Carolina at Chapel Hill (USA) and the Chinese Center for Disease Control and Prevention. This study followed Strengthening the Reporting of Observational Studies in Epidemiology (STROBE) reporting guidelines.

For the present study, we considered 1997 as baseline year based on the availability of complete dietary intake information and comprehensive covariates. The exclusion criteria are as follows: (1) diagnosis of memory-related disease at baseline (Alzheimer’s disease or dementia); (2) died within the first 2 years of follow-up to reduce reverse causation; (3) with implausible total energy intakes at baseline (< 500 or > 3500 kcal/day in female and < 800 or > 4000 kcal/day in male according to nutritional epidemiology protocols to exclude reporting bias [34]); (4) missing data for covariates at baseline; (5) age < 60 years since age group of 60 and over are more likely to have cognitive decline. A total of 3404 participants with valid data were included in the current study for subsequent analysis (Supplementary Fig. 1).

### Dietary assessment and PHDI calculation

In the current study, dietary intake was assessed in 1997, 2000, 2004, 2006, 2009, 2011 as the 2015 survey data are not publicly available. At each wave, trained researchers collected individual-level dietary data using three consecutive 24-hour dietary recalls, in combination with household-level food inventory weighing during the same period (2 weekdays and 1 weekend day). This method previously validated in detail reduces day-to-day variation and improves accuracy in capturing usual intake [35]. Specifically, all foods and condiments were measured using Chinese balance scales (graduation:10 g), recorded with traditional Chinese mass unit (JIN). The 3-day average intake was calculated for each wave and converted into standardized food group intakes based on the Chinese Food Composition Table (2002 version and 2004 version). The USDA database of 2005 was used to calculate added sugars owing to the lack of available data in the Chinese Food Composition Database. All dietary components were quantified as grams per day (g/day) using standardized conversion protocols. To account for within-person variation and better represent habitual intake, cumulative average daily intake across waves was computed from baseline to the last visit.

The PHDI was calculated to estimate the adherence to the EAT-Lancet Commission recommendations for healthy and environmentally diets within sustainable food systems [19]. Comprising 14 EAT-Lancet food groups categorized as recommended or restricted based on sustainability criteria, the PHDI scoring system reflects dual health-environment objectives. Each food group receives 0–10 points through quantitative benchmarking against EAT-Lancet reference values, where higher scores indicate closer adherence to optimal intake ranges. In brief, the PHDI was standardized based on the energy intake of individual to 2500 kcal/d, with component-specific reference intakes established in prior methodological studies [36] (Supplementary Table 1). Each energy-adjusted food group was assigned PHDI cutoff, consumption in between the minimum and maximum thresholds are scored proportionally according the continuous scoring system. The individual scores for each food group are then summed to obtain the participant’s total score, which ranged from 0 (non-adherence) to 140 points (full adherence).

### Outcomes

The primary outcome was a cognitive score summed from the cognitive screening items used in CHNS. Cognitive performance was evaluated using an adapted Chinese version of the Telephone Interview for Cognitive Status (TICS-m), administered by certified interviewers across five survey waves (1997–2006) [37]. The cognition screening included immediate and delayed recall of a 10-word list (10 points each), counting backward from 20 (2 points), and serial 7 subtraction (5 points). The total score across all items ranged from 0 to 27 with higher scores indicating better cognitive function. Z-score standardization was implemented across survey waves (reference: baseline measurements) to ensure comparability of three cognitive domains: verbal memory, attention, and calculation ability. A verbal memory Z-score was created for each wave by averaging the Z-scores from the immediate and delayed recall tests at each wave, an attention Z-score by averaging the Z-scores from counting backward from 20 at each wave, a calculation Z-score by averaging the Z-scores from serial 7 subtraction at each wave, and a global cognitive score by averaging the Z-scores from all the cognitive tests at each wave. A positive Z-score indicates better cognitive function than the mean population score, and a negative score indicates a poor cognitive score.

### Covariates

At baseline, data on these possible covariates to demographic characteristics (age, sex, BMI, marital status, urban or rural residence and administrative regions), socio-economy status (education, monthly per capita income/Yuan, ), lifestyle behaviors (current smoking, current drinking and physical activity), and physical health (hypertension, diabetes) were obtained from the CHNS. Body mass index (BMI) was calculated as the measured weight (in kg) divided by the squared measured height (in meters). BMI categories were divided into ≤ 18.5 kg/ m^2^, 18.5–24.9 kg/m^2^, 25.0–29.9 kg/m^2^, ≥ 30 kg/m^2^ according to the World Health Organization classification. Marital status was dichotomized as married vs. unmarried (encompassing widowed, divorced, separated, or never married) through structured interviews. We clustered specific provinces based on their administrative regions including Northern China, Eastern China, Central China and Southern China. Education was classified as primary level (1–6 years of schooling or below), middle level (middle school) and high level (high school or above).

Physical activity (PA) was assessed at baseline using a modified short-form International Physical Activity Questionnaire (IPAQ), which captured domain-specific energy expenditure in four areas: transportation, domestic work, occupational activity, and leisure-time exercise. Participants reported the frequency and duration of each activity, from which weekly total metabolic equivalent task (MET) were calculated. Participants were categorized based on the highest intensity of activity they reported. Vigorous PA included activities that make breathing much harder than normal (e.g. heavy lifting, plowing, aerobics, fast cycling); moderate PA included activities that make breathing somewhat harder (e.g. brisk walking, regular cycling, mopping, Tai Chi); light PA included walking at work or home, for commuting, or for recreation. This classification aligns with the WHO physical activity guidelines [38].

### Statistical analysis

For baseline information, we used mean ± SD or median (IQR) for continuous variables and n (%) for categorical variables. Participants were stratified into quintiles based on cumulative PHDI scores, with the first quintile serving as the reference group. In the primary analysis, we assessed the association between adherence to the cumulative average PHDI and cognitive decline using linear mixed model with random intercepts and slope. Age at each survey wave was parameterized as the time metric to control for chronological aging effects. The interaction term between PHDI adherence quintiles and age was included in each model to evaluate the longitudinal association between adherence to the PHDI and cognitive decline over time. Trend analyses across quintiles were performed by assigning participants the median value of each quintile and assessing the continuous association. We ran two adjusted models: model 1 was adjusted baseline age, gender, education, marriage, and residency, region, and household income per capita; and model 2 was adjusted for model 1 plus smoking, alcohol consumption, and BMI category. Model 2 is considered the main model in our analyses. To examine sex, BMI, smoking status, alcohol intake status, urban areas, PA, and administrative regions as potential effect modifiers of the association between adherence to the PHDI and cognitive decline, we used a three-way interaction term among the quintiles of PHDI adherence, the age and potential effect modifiers on the fully adjusted model. We evaluated heterogeneity in significant interaction by computing the differences of cognitive decline obtained from potential confounding adjustment subgroup in stratified analyses. Adjustment variables serving as stratification factors were excluded from corresponding regression models to avoid multicollinearity.

To test the robustness of our findings, we conducted five sensitivity analyses. First, we repeated the main analysis after additionally adjustment for hypertension, diabetes, cardiovascular disease, daily socialization and total calories. Second, we also verified our analysis using each wave as the slope of time to assess the consistency of our results. Third, to further explore the different role of score construction, we reran the main analysis by adopting a previous strategy by defined a PHDI ranging from 0 to 14 points [39]. Fourth, we further analyzed these associations by imputing covariates described above using multiple imputation by chained equations (mice package) that incorporated all variables in this study.

All statistical analyses were performed using the R (version 4.4.1 software). All statistical tests were two sided, and *P* < 0.05 was considered statistically significant.

## Results

### Baseline characteristics

In this study, we included a total of 3404 participants as final baseline sample, among whom the mean age was 65.2 (SD 5.8) years, and 1597 (46.9%) were male (Table 1). Among them, 78.7% married, 37.4% lived in urban areas, 31.9% and 30.1% were current smokers and alcohol drinkers, respectively. The median (interquartile range, IQR) duration of follow-up was 12 (7–14) years. The mean PHDI scores ranged from 66.2 (SD 5.5) to 85.6 (SD 5.9) in the first versus fifth quantile PHDI adherence in the CHNS. The mean PHDI scores increased from 72.7 (SD 5.7) in 1997 to 74.4 (SD 9.1) in 2011, indicating only limited changes in dietary habits over the study period (Supplementary Table 2). At baseline, participants who scored on the fifth quantile of PHDI adherence tended to have more energy intake, higher education level, be women, live in central administrative regions compared to participants on the first quantile. Participants on the fifth quantile of PHDI adherence were also more likely to have a healthier lifestyle (less likely to be smokers, less likely to be alcohol drinkers, more physical activity) compared to participants classified as on the first quantile.

**Table 1:** Descriptive statistics of the study participants at baseline by quintiles of the PHDI adherence score

	Overall *N* = 3404	Quintile 1 *N* = 681	Quintile 2 *N* = 681	Quintile 3 *N* = 681	Quintile 4 *N* = 681	Quintile 5 *N* = 680	*P*
PHDI	73.6 (7.5)	66.2 (5.5)	70.5 (0.1)	71.2 (0.3)	74.7 (1.9)	85.6 (5.9)	0.000
Age	65.2 (5.8)	64.9 (5.8)	65.6 (5.8)	65.4 (5.9)	65.0 (5.7)	65.0 (5.9)	0.057
Energy intake, kcal/d	2080.9 (635.7)	2046.7 (649.5)	2121.6 (645.6)	2022.2 (611.0)	2079.1 (611.9)	2135.1 (653.8)	0.004
BMI	23.1 (3.6)	23.2 (3.7)	22.7 (3.6)	23.0 (3.6)	23.5 (3.6)	23.4 (3.6)	<0.001
Male (%)	1597 (46.9%)	322 (47.3%)	329 (48.3%)	291 (42.7%)	338 (49.6%)	317 (46.6%)	0.116
Married (%)	2679 (78.7%)	547 (80.3%)	520 (76.4%)	532 (78.1%)	542 (79.6%)	538 (79.1%)	0.430
Education							<0.001
Primary	2529 (74.3%)	504 (74.0%)	530 (77.8%)	538 (79.0%)	472 (69.3%)	485 (71.3%)	
Middle	444 (13.0%)	97 (14.2%)	87 (12.8%)	70 (10.3%)	96 (14.1%)	94 (13.8%)	
High	431 (12.7%)	80 (11.7%)	64 (9.4%)	73 (10.7%)	113 (16.6%)	101 (14.9%)	
Urban (%)	1274 (37.4%)	234 (34.4%)	214 (31.4%)	269 (39.5%)	293 (43.0%)	264 (38.8%)	<0.001
Region (%)							<0.001
Northern	587 (17.2%)	143 (21.0%)	88 (12.9%)	83 (12.2%)	139 (20.4%)	134 (19.7%)	
Eastern	850 (25.0%)	161 (23.6%)	145 (21.3%)	183 (26.9%)	198 (29.1%)	163 (24.0%)	
Central	1065 (31.3%)	194 (28.5%)	246 (36.1%)	217 (31.9%)	188 (27.6%)	220 (32.4%)	
Southern	902 (26.5%)	183 (26.9%)	202 (29.7%)	198 (29.1%)	156 (22.9%)	163 (24.0%)	
Household income per capita, Yuan	5753.3 (7828.3)	6107.1 (8521.4)	4540.1 (6019.5)	4694.7 (6081.2)	6626.7 (7021.2)	6799.2 (7202.5)	<0.001
Smoking (%)	1085 (31.9%)	237 (34.8%)	228 (33.5%)	206 (30.2%)	220 (32.3%)	194 (28.5%)	0.096
Current drinkers (%)	1025 (30.1%)	220 (32.3%)	177 (26.0%)	214 (31.4%)	211 (31.0%)	203 (29.9%)	0.096
Hypertension (%)	635 (18.7%)	136 (20.0%)	101 (14.8%)	124 (18.2%)	131 (19.2%)	143 (21.0%)	0.039
Diabetes (%)	130 (3.8%)	24 (3.5%)	24 (3.5%)	25 (3.7%)	30 (4.4%)	27 (4.0%)	0.903
Physical activity (%)							0.264
Light	1818 (58.8%)	337 (55.8%)	360 (61.0%)	391 (61.8%)	361 (56.5%)	369 (58.9%)	
Moderate	677 (21.9%)	134 (22.2%)	118 (20.0%)	133 (21.0%)	146 (22.8%)	146 (23.3%)	
Vigorous	598 (19.3%)	133 (22.0%)	112 (19.0%)	109 (17.2%)	132 (20.7%)	112 (17.9%)	

### Association between PHDI adherence and cognitive decline

Over the follow-up period, the overall rates of cognitive decline in the total population varied by domain. The average rate of decline was − 0.047 standard deviation units (SDU) per year for memory (95% CI: -0.054 to -0.041), -0.036 SDU/year for attention (95% CI: -0.043 to -0.028), and − 0.027 SDU/year for global cognition (95% CI: -0.031 to -0.023), indicating progressive age-related declines in these domains. In contrast, calculation abilities showed a slight, non-significant decrease over time, with an estimated change of -0.009 SDU/year (95% CI: -0.016 to 0.003).

**Fig. 1:**
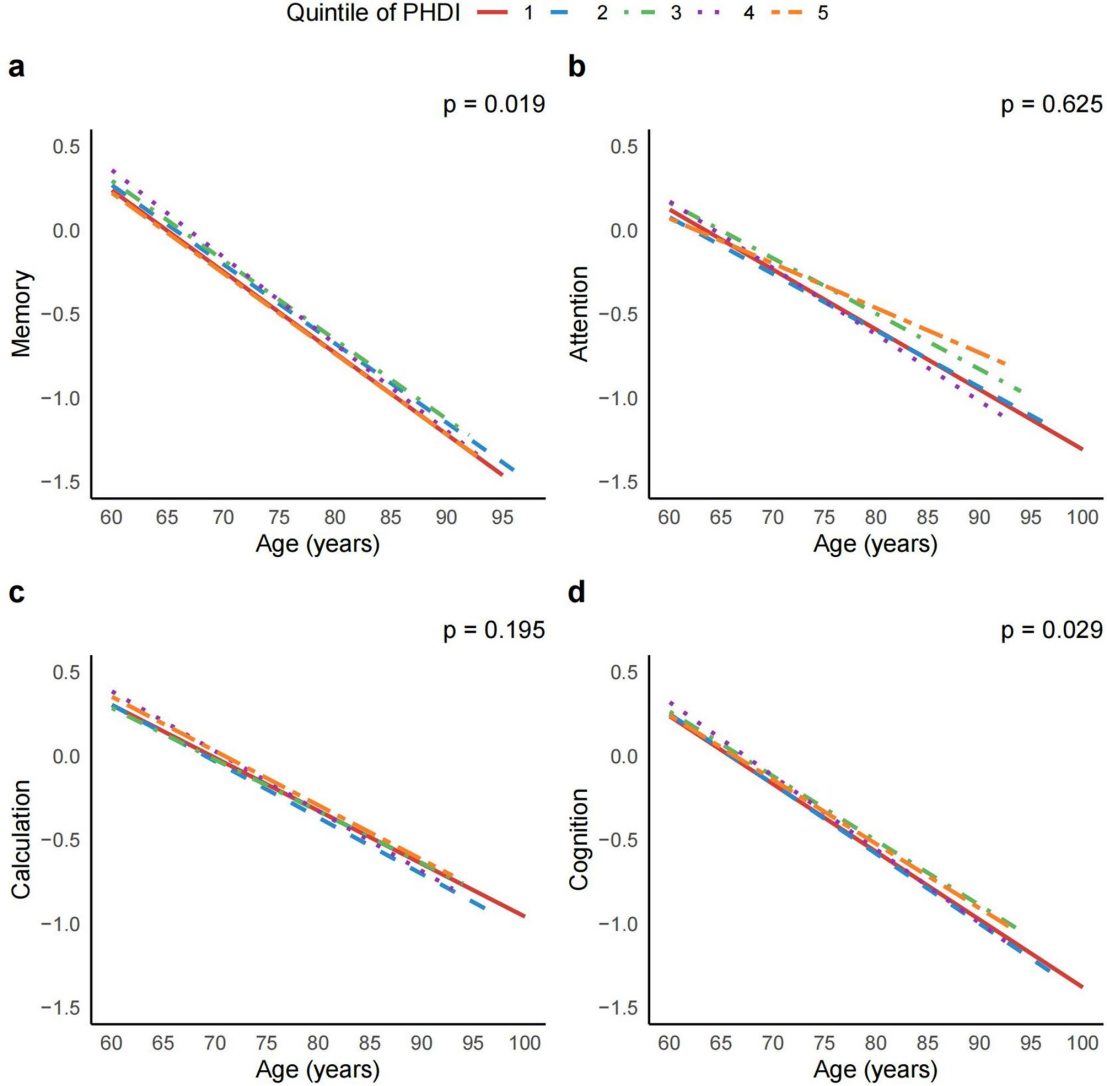
Associations between quantiles of the PHDI adherence and cognition decline. Associations between quantile the cumulative average PHDI adherence scores and memory (**a**), attention (**b**), calculation (**c**) and global cognition (**d**) over a median of 12 years of follow-up. Linear mixed-effects model adjusted for age, gender, education, marriage, and residency, region, household income per capita, smoking, alcohol consumption, and BMI category (*n* = 3404). *P* value represents the *P* for trend of the interaction term between quintiles of PHDI and age, calculated by the Wald test (two-sided)

Adherence to the PHDI was associated with slower memory and global cognitive decline. In the unadjusted model, participants in the highest quintile of PHDI adherence had significantly slower memory decline (B = 0.030, 95%CI = 0.005–0.056, *P* = 0.021) and global cognitive decline (B = 0.025, 95%CI = 0.007–0.042, *P* = 0.005) compared with those in the lowest quintile, with a significant linear trend across quintiles (memory: *P* for trend = 0.033, global cognition: *P* for trend = 0.023; Fig. 1, Supplementary Table 3). These associations remained significant after adjusting for demographic, lifestyle, and medical covariates. In model 2, memory decline was slower in the fifth quintile (B = 0.025, 95%CI = 0.000-0.049, *P* = 0.049) and global cognition decline was attenuated (B = 0.020, 95%CI = 0.004–0.037, *P* = 0.016) compared with the lowest quintile, with consistent trend relationships (memory: *P* for trend = 0.019, global cognition: *P* for trend = 0.029; Fig. 1, Supplementary Table 3). No significant associations were observed between PHDI adherence and decline in attention or calculation abilities (Fig. 1, Supplementary Table 3). Similar inverse associations between the PHDI adherence and cognitive decline were observed in further analyses, including additional adjustments for hypertension, diabetes, cardiovascular disease, daily socialization and total calories (Supplementary Table 4), using each wave as the slope of time (Supplementary Table 5), imputing covariates described above (Supplementary Table 7). After adopting a strategy by defined a PHDI ranging from 0 to 14 points, sensitivity analysis in the linear mixed model yielded consistent results (Supplementary Table 6).

**Fig. 2:**
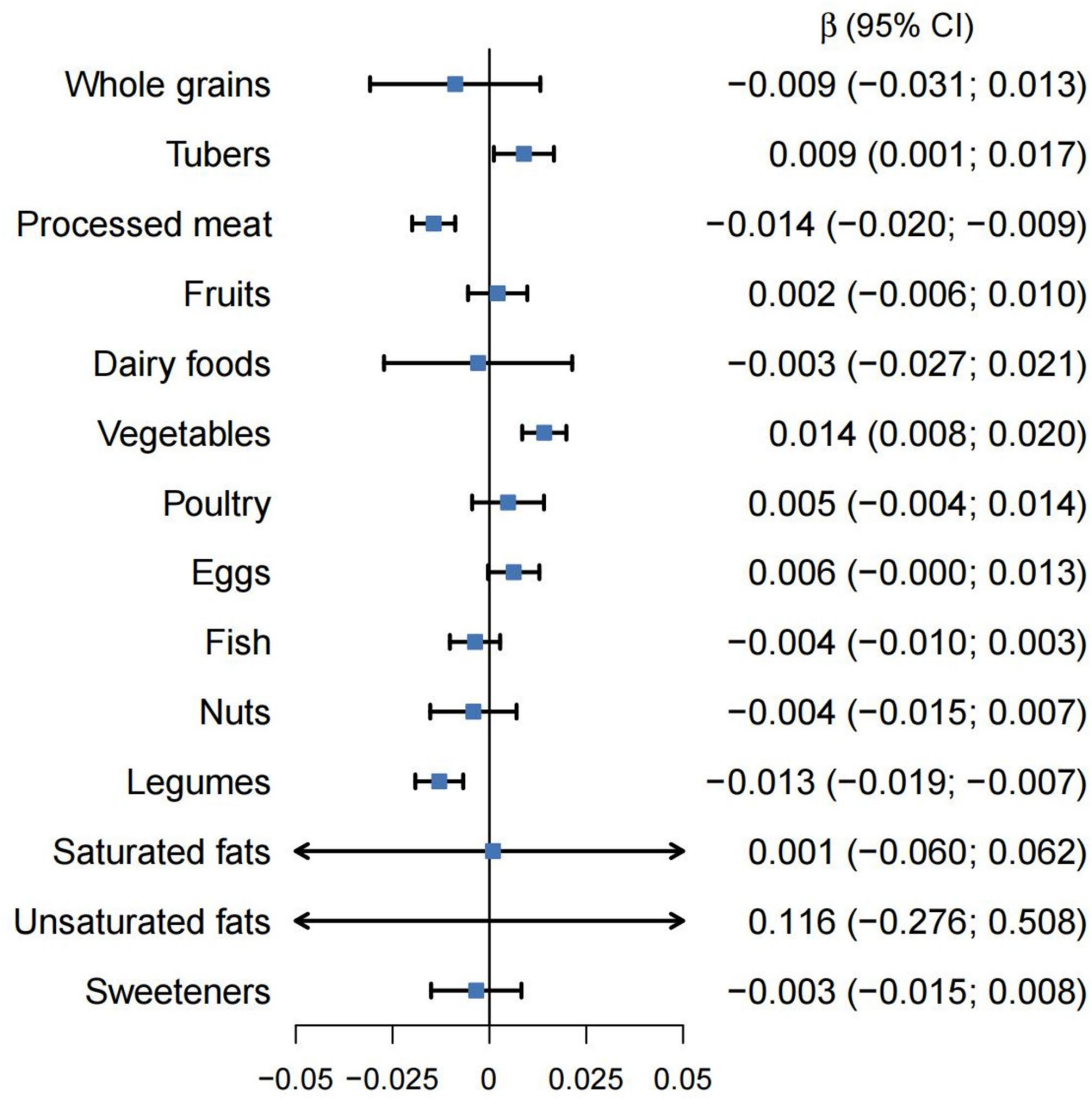
Association between adherence to the individual PHDI food components and global cognition decline. Estimates, 95% CIs and *P* values were derived from linear mixed-effects model adjusted for age, gender, education, marriage, and residency, region, household income per capita, smoking, alcohol consumption, and BMI category (*n*=3404). The data are presented as mean estimates and 95% CIs

We further estimated the associations between each of the 14 PHDI components and global cognition decline. Participants with lower intake of legumes and soy foods and higher intake of red and processed meat experienced faster global cognitive decline. Higher adherence to the tubers and vegetables was associated with slower global cognitive decline (Fig. 2).

### Physical activity as an effect modifier

Our results indicated that PA was an effect modifier in the association between the PHDI adherence and cognitive decline in the memory and global cognition domain. Participants on the fifth quantile of PHDI adherence who engaged in vigorous PA exhibited an attenuated annual memory and global cognitive decline(memory: B = 0.070, 95%CI 0.010–0.130, *P* = 0.022, *P* for trend = 0.013; global cognition: B = 0.045, 95%CI 0.003–0.086, *P* = 0.034, *P* for trend = 0.037) compared to those who had moderate (memory: B=-0.036, 95%CI -0.089-0.016, *P* = 0.175, *P* for trend = 0.750; global cognition: B=-0.011, 95%CI -0.046-0.024, *P* = 0.525, *P* for trend = 0.234) and light PA (memory: B = 0.026, 95%CI 0.009–0.061, *P* = 0.043, *P* for trend = 0.005; global cognition: B = 0.028, 95%CI 0.005–0.051, *P* = 0.016, *P* for trend = 0.101), respectively. There are no associations between adherence to the PHDI and attention and calculation cognition domains in participants with moderate PA (Table 2). Although statistical significance varied across different PHDI definitions in the sensitivity analysis, the modifying effect of PA in annual cognitive decline remained consistently evident (Supplementary Table 8). We found no evidence of effect modification by sex, BMI, smoking status, alcohol intake status, urban areas, and administrative regions.

**Table 2:** Association between quintiles of adherence to the cumulative average PHDI and annual cognitive decline stratified according to physical activity

	Light (*n* = 1818)		Moderate (*n* = 677)		Vigorous (*n* = 598)	
	β (95% CI)	*P*	β (95% CI)	*P*	β (95% CI)	*P*
**Memory**						
Quintile 1 * age	Reference		Reference		Reference	
Quintile 2 * age	0.024 (-0.006; 0.055)	0.112	-0.030 (-0.078; 0.018)	0.216	0.004 (-0.048; 0.055)	0.882
Quintile 3 * age	0.039 (0.009; 0.069)	0.011	0.055 (0.007; 0.102)	0.023	0.016 (-0.035; 0.068)	0.537
Quintile 4 * age	0.033 (0.001; 0.064)	0.043	0.010 (-0.038; 0.059)	0.675	0.061 (0.004; 0.117)	0.037
Quintile 5 * age	0.026 (0.009; 0.061)	0.043	-0.036 (-0.089; 0.016)	0.175	0.070 (0.010; 0.130)	0.022
*P* for trend		0.005		0.750		0.013
**Attention**						
Quintile 1 * age	Reference		Reference		Reference	
Quintile 2 * age	0.015 (-0.019; 0.050)	0.374	-0.035 (-0.090; 0.020)	0.209	0.006 (-0.056; 0.067)	0.854
Quintile 3 * age	0.021 (-0.013; 0.055)	0.230	-0.002 (-0.056; 0.053)	0.955	0.068 (0.007; 0.130)	0.029
Quintile 4 * age	0.015 (-0.021; 0.050)	0.414	-0.029 (-0.086; 0.027)	0.307	0.034 (-0.034; 0.101)	0.328
Quintile 5 * age	0.014 (-0.026; 0.053)	0.495	-0.009 (-0.070; 0.051)	0.762	0.035 (-0.037; 0.106)	0.345
*P* for trend		0.565		0.432		0.322
**Calculation**						
Quintile 1 * age	Reference		Reference		Reference	
Quintile 2 * age	-0.004 (-0.033; 0.025)	0.796	-0.003 (-0.050; 0.044)	0.890	0.023 (-0.033; 0.078)	0.427
Quintile 3 * age	-0.005 (-0.034; 0.024)	0.738	-0.031 (-0.077; 0.016)	0.193	0.044 (-0.012; 0.100)	0.121
Quintile 4 * age	0.003 (-0.037; 0.010)	0.853	-0.030 (-0.078; 0.019)	0.228	-0.004 (-0.066; 0.057)	0.894
Quintile 5 * age	0.044 (0.010; 0.078)	0.011	0.003 (-0.048; 0.055)	0.900	0.027 (-0.038; 0.092)	0.417
*P* for trend		0.089		0.201		0.398
**Cognition**						
Quintile 1 * age	Reference		Reference		Reference	
Quintile 2 * age	0.011 (-0.008; 0.031)	0.260	-0.025 (-0.057; 0.007)	0.121	0.011 (-0.024; 0.046)	0.539
Quintile 3 * age	0.018 (-0.002; 0.037)	0.077	-0.028 (-0.059; 0.004)	0.085	0.043 (0.008; 0.079)	0.017
Quintile 4 * age	0.016 (-0.005; 0.036)	0.127	-0.016 (-0.049; 0.017)	0.343	0.030 (-0.009; 0.069)	0.131
Quintile 5 * age	0.028 (0.005; 0.051)	0.016	-0.011 (-0.046; 0.024)	0.525	0.045 (0.003; 0.086)	0.034
*P* for trend		0.101		0.234		0.037

B, estimate; CI, confidence interval

Estimate, confidence intervals, and p-values were calculated using linear mixed-effects models (two-sided)

The Linear mixed-effects model adjusted for age, s gender, education, marriage, and residency, region, household income per capita, smoking, alcohol consumption, and BMI category

*P* for trend using the Wald test

## Discussion

In this prospective cohort study of 3404 Chinese older adults with normal cognitive function at baseline, we observed an inverse association between planetary health reference diet, as measured by the Planetary Health Diet Index, and cognitive decline over 12 years of follow-up. Regarding specific cognitive domains, individuals with higher adherence to the PHDI exhibited better memory functioning and slower annual decline. No significant associations were observed for attention or calculation abilities. The associations were significantly modified by PA, highlighting that vigorous PA is an important preventive intervention against cognitive decline in participants aged 60 years or older. Analysis of individual component of PHD revealed that higher consumption of tubers, all vegetables, lower intake of legumes and soy foods was associated with slower global cognitive decline. A set of sensitivity analyses confirmed the robustness of our findings. Our results suggest that higher adherence to the dietary pattern might be dual beneficial to environmental sustainability and cognitive function in older adults.

Despite the widespread academic discussion on this diet, few studies so far have investigated the associations with cognitive function, particularly in China. To the best our knowledge, limited observational studies have documented the relations between PHDI and cognitive health. Gonçalves et al. using data from the ELSA-Brasil cohort reported that higher adherence to the PHDI was associated with slower memory decline and global cognitive health [25]. In the Singapore Chinese Health Study, a higher one-SD increase in PHDI score was associated with an 11% slower risk of poor cognitive function in non-carriers of APOE ε4 allele [26]. However, as this study was conducted in Singapore, a high-income country with specific sociodemographic and healthcare contexts may have limited generalizability to LMICs where dietary, environmental, and disease profiles differ substantially. In the UK Biobank cohort of over 190,000 participants, Zhao et al. found an inverse association between adherence to the PHD and all-cause dementia, as well as later-onset dementia for individuals with high socioeconomic status level over 12 years of follow-up [40]. However, their use of a simple binary scoring system (awarding 1 point for each recommendation that was met and 0 points if it was not met) may have limited the ability to distinguish degrees of adherence. Conversely, a previous cross-sectional study found no association between adherence to the PHD and cognitive performance [31]. This discrepancy may be due to the relatively smaller sample size (*n* = 328) and younger adults (18–35 years old) in this study. In contrast, our study population primarily consisted of older adults at baseline (mean age, 65.2) and followed up 12 years at later life. Similar to our study, investigators of a community-dwelling adults aged ≥ 65 years using a tertiary score found greater adherence to the PHD was associated with better cognitive functioning (*β* [95% CI]: 0.04 [0.00, 0.08]) and slower rate of decline (*β* [95% CI]: 0.05 [0.02, 0.08]) [28].

There is scarce evidence investigating the association between PHD and cognitive decline across PA, despite PA as a significant protective factor in reducing dementia and AD risk [41, 42]. In our study, PA levels were classified as light, moderate, or vigorous according to total weekly MET, derived by multiplying the self-reported duration of each activity by its assigned MET value based on the IPAQ [43]. Our findings indicate that the association between adherence to the PHDI and cognitive function varies by PA, with vigorous PA conferring potential beneficial effect on slower memory and global cognitive decline, whereas not shown in moderate PA.

Emerging evidence showed that PA has gained concern in other dietary patterns. Using data from the Health and Retirement Study, Ahn et al. revealed that higher adherence to the MIND diet was associated with better global cognition and a lower risk of cognitive decline in participants with high-intensity PA compared to neither behavior [44]. In addition, a study of 132 participants from the UCSF Memory and Aging Center’s Longitudinal Brain Aging Program found that higher levels of PA associated with better executive functioning and gray matter volume, particularly when diet is poor [45]. In the Memory and Aging Project with 215 community-dwelling older adults, the cognitive benefits of PA were more pronounced in individuals with lower adherence to the MIND diet [46]. However, a previous study from the Longitudinal Aging Study Amsterdam (LASA) cohort did not identify a significant interaction among sufficient PA, healthy diet adherence and good cognitive function [47]. Nonetheless, the LASA study did report that both sufficient daily PA (OR [95% CI]: 2.545 [2.006, 3.228]) and adherence to a healthy diet (OR [95% CI]: 1.766 [1.224, 2.546]) were independently associated with good global cognition. The discrepancy in findings may be attributed to differences in PA classification, as the LASA study used a dichotomous threshold (≥ 20 min/day of moderate-intensity activity), whereas our study employed a tripartite stratification (light, moderate, vigorous), potentially influencing outcomes. The interaction between specific dietary patterns and PA, whether individually or in combination, in relation to cognitive function in older adults has received considerable attention in recent reviews [48, 49]. Although PA demonstrates therapeutic potential in alleviating depressive or anxiety symptoms, enhancing neuroprotective effects, and improving overall well-being, the prevalent PA among older adults may account for its pronounced modifying effects on cognitive outcomes in this population.

Individual components of the PHDI exhibited distinct associations with global cognitive decline. Higher consumption of tubers, vegetables, and legumes and soy foods was associated with slower global cognitive decline, whereas adherence to high consumption of processed meat was associated with worse cognitive performance. A recent review reported that higher consumption of total soy products is associated with reduced cognitive impairment risk, while tofu intake has an inverse relationship with cognitive performance. The strongest neuroprotective associations emerged specifically with increased soybean consumption, suggesting that the cognitive benefits of soy products may be source-dependent, likely due to variations in bioactive compounds [50]. Vegetables, as the emphasized component of mainly plant-based dietary patterns, demonstrate well-documented inverse associations with cognitive decline, which was supported by the previous study using summary data of the CLHLS [51] and NHANES [52]. Antoneta et al. using data from 1921 birth cohort from Northeast United Kingdom found that high consumption of red meat, potato, and gravy are associated with poor cognitive functioning but not with rate of cognitive decline in very older adults. Such inconsistencies may stem from differences in study populations, design and outcome measures. A critical consideration is that food groups are rarely consumed in isolation but rather within complex dietary matrices, where nutrients interact in dynamic synergistic interplay [53, 54]. Future studies are needed to assess the potential health effects of PHDI in older adults across different countries within a comparative framework.

The strengths of this study included the prospective study design and the large sample size, but several limitations should be acknowledged. First, although we excluded individuals with cognitive impairment or dementia at baseline to reduce the possibility of reverse causality, residual confounding and reverse causation cannot be entirely ruled out, which limits causal inference between adherence to the cognitive health. Second, diet is a time-varying exposure and our study had limited ability to capture dietary changes over time. Nonetheless, while measurement error in dietary assessment is inevitable, the use of multiple 3-day dietary recalls may help mitigate misreporting bias. Third, the cognitive function scores used in this study were derived from the TICS-m, which was administered only in the 1997, 2000, 2004, and 2006. After 2006, CHNS ceased the collection of cognitive function data. In contrast, dietary data were available for 1997, 2000, 2004, 2006, 2009, and 2011, with three consecutive 24-hour recalls discontinued in waves after 2011. This led to a mismatch in the temporal span of exposure and outcome data. Finally, although our study encompasses a broad geographic range within China, the global generalizability and internal validity of our findings need further validation in other cohort studies.

## Conclusion

In conclusion, this prospective study provides evidence to support higher adherence to the planetary health reference diet, reflected by PHDI, was associated with slower cognitive decline in older adults. Further studies are needed to assess the link between the planetary health reference diet and cognition by which physical activity may be an effect modifier in diverse populations.

## Electronic supplementary material

Below is the link to the electronic supplementary material.

Supplementary Material 1

## Data Availability

Data used for this study, including individual participant data and data dictionaries, are publicly available on the CHNS website (https://www.cpc.unc.edu/projects/china).
