# Supplementary material for: Adherence to the planetary health diet is associated with slower cognitive decline: a prospective cohort analysis of Chinese older adults
Source: Int J Behav Nutr Phys Act. 2025 May 16;22:56. doi: 10.1186/s12966-025-01759-y (PMC12082918; doi:10.1186/s12966-025-01759-y)
Supplement: Supplementary file 1 — Supplementary Material 1 [file 12966_2025_1759_MOESM1_ESM.docx]

**Supplemental materials:**

**Supplementary Table 1 Calculation of the Planetary Health Diet lndex Scores.**

**Supplementary Table 2 Planetary Health Diet lndex Scores by timepoint in China Health and Nutrition Survey.**

**Supplementary Table 3 Association between quintiles of the Planetary Health Diet Index Scores at the study baseline and annual cognitive decline.**

**Supplementary Table 4 Sensitivity analysis on the associations between quintiles of the Planetary Health Diet Index Scores at the study baseline and annual cognitive decline after further adjustment for hypertension, diabetes, cardiovascular disease, daily socialization and total calories.**

**Supplementary Table 5 Sensitivity analysis on the associations between quintiles of the Planetary Health Diet Index Scores at the study baseline and annual cognitive decline using time from waves as timescale.**

**Supplementary Table 6 Sensitivity analysis on the associations between quintiles of the Planetary Health Diet Index Scores* at the study baseline and annual cognitive decline.**

**Supplementary Table 7 Sensitivity analysis on the associations between quintiles of the Planetary Health Diet Index Scores at the study baseline and annual cognitive decline by using the dataset with imputation.**

**Supplementary Table 8 Sensitivity analysis on the associations between quintiles of the Planetary Health Diet Index Scores* at the study baseline and annual cognitive decline stratified according to physical activity.**

**Supplementary Figure 1. Flow chart of study population.**

| **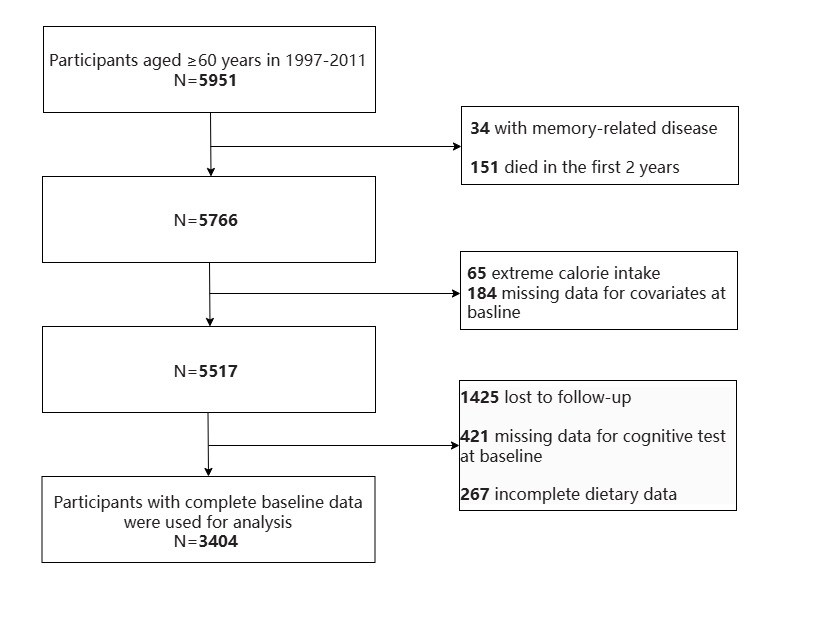** |
| --- |
| **Supplementary Figure 1. Flow chart of study population.** |

**Supplemental Table 1. Calculation of the Planetary Health Diet lndex Scores.**

| Type | Dietary components | EAT-Lancet Reference diet (g/2500 kcal) | PHDI scoring criteria (total 0–140) | |
| --- | --- | --- | --- | --- |
|  |  |  | Min score (0) in g/day | Max score (10) in g/day |
| Recommended | Whole grains | 232 (0–464) | 0 | 232 |
| Limited | Tubers | 50 (0–100) | ≥200 | ≤50 |
| Recommended | All vegetables | 300 (200–600) | 0 | ≥300 |
| Recommended | Fruits | 200 (100–300) | 0 | ≥200 |
| Limited | Dairy foods | 250 (0–500) | ≥1000 | ≤250 |
| Limited | Red or processed meat | 14 (0–28) | ≥100 | ≤14 |
| Limited | Poultry | 29 (0–58) | ≥100 | ≤29 |
| Limited | Eggs | 13 (0–25) | ≥120 | ≤13 |
| Recommended | Fish | 28 (0–100) | 0 | ≥28 |
| Recommended | Nuts | 50 (0–75) | 0 | ≥50 |
| Recommended | Legumes and soy foods | 75 (0–150) | 0 | ≥150 |
| Limited | Saturated fats | 11.8 (0–11.8) | ≥10% of total energy intake | 0% of total energy intake |
| Recommended | Unsaturated fats | 40 (20–80) | ≤3.5% of total energy intake | ≥21% of total energy intake |
| Limited | All sweeteners | 31 (0–31) | ≥25% of total energy intake | ≤5% of total energy intake |

The EAT-Lancet reference diet recommendations are designed based on a total energy intake of 2500 kcal/day

**Supplemental Table 2. Planetary Health Diet lndex Scores by timepoint in China Health and Nutrition Survey.**

| Year | Mean (SD) | Median (IQR) |
| --- | --- | --- |
| 1997 | 72.7 (5.7) | 70.9 (70.4, 72.0) |
| 2000 | 72.6 (5.6) | 71.0 (70.5, 72.7) |
| 2004 | 74.4 (8.7) | 71.7 (70.3, 79.3) |
| 2006 | 74.6 (8.8) | 71.8 (70.3, 79.3) |
| 2009 | 74.7 (8.6) | 71.8 (70.4, 79.9) |
| 2011 | 74.4 (9.1) | 71.9 (70.3, 80.0) |

**Supplementary Table 3 Association between quintiles of adherence to the cumulative average PHDI and annual cognitive Decline.**

|  | **Unadjusted** | | **Model 1** | | **Model 2** | |
| --- | --- | --- | --- | --- | --- | --- |
|  | **β (95% CI)** | ***P*** | **β (95% CI)** | ***P*** | **β (95% CI)** | ***P*** |
| **Memory** |  |  |  |  |  |  |
| Quintile 1 * age | Reference | | Reference | | Reference | |
| Quintile 2 * age | 0.008 (-0.014; 0.031) | 0.459 | 0.010 (-0.012; 0.031) | 0.375 | 0.010 (-0.011; 0.031) | 0.362 |
| Quintile 3 * age | 0.011 (-0.012; 0.033) | 0.347 | 0.011 (-0.010; 0.033) | 0.308 | 0.013 (-0.009; 0.034) | 0.253 |
| Quintile 4 * age | 0.026 (0.002; 0.050) | 0.033 | 0.027 (0.004; 0.050) | 0.019 | 0.027 (0.005; 0.050) | 0.018 |
| Quintile 5 * age | 0.030 (0.005; 0.056) | 0.021 | 0.025 (0.001; 0.050) | 0.045 | 0.025 (0.000; 0.049) | 0.049 |
| *P* for trend |  | 0.033 |  | 0.029 |  | 0.019 |
| **Attention** |  | | | | | |
| Quintile 1 * age | Reference | | Reference | | Reference | |
| Quintile 2 * age | -0.003 (-0.028; 0.023) | 0.844 | 0.000 (-0.025; 0.025) | 0.975 | 0.000 (-0.025; 0.025) | 0.996 |
| Quintile 3 * age | 0.014 (-0.012; 0.040) | 0.289 | 0.016 (-0.009; 0.041) | 0.215 | 0.017 (-0.008; 0.042) | 0.192 |
| Quintile 4 * age | -0.007 (-0.034; 0.020) | 0.601 | -0.003 (-0.029; 0.024) | 0.851 | -0.002 (-0.028; 0.024) | 0.887 |
| Quintile 5 * age | 0.016 (-0.014; 0.045) | 0.295 | 0.011 (-0.018; 0.040) | 0.463 | 0.011 (-0.018; 0.040) | 0.450 |
| *P* for trend |  | 0.823 |  | 0.903 |  | 0.625 |
| **Calculation** |  | | | | | |
| Quintile 1 * age | Reference | | Reference | | Reference | |
| Quintile 2 * age | -0.007 (-0.029; 0.015) | 0.520 | -0.007 (-0.028; 0.015) | 0.540 | -0.007 (-0.028; 0.015) | 0.545 |
| Quintile 3 * age | 0.006 (-0.028; 0.016) | 0.613 | 0.005 (-0.026; 0.017) | 0.661 | 0.004 (-0.026; 0.017) | 0.684 |
| Quintile 4 * age | -0.014 (-0.037; 0.010) | 0.254 | -0.010 (-0.033; 0.012) | 0.365 | -0.010 (-0.033; -0.033) | 0.365 |
| Quintile 5 * age | 0.028 (0.003; 0.054) | 0.028 | 0.024 (-0.001; 0.048) | 0.059 | 0.024 (-0.001; 0.048) | 0.057 |
| *P* for trend |  | 0.224 |  | 0.154 |  | 0.195 |
| **Cognition** |  | | | | | |
| Quintile 1 * age | Reference | | Reference | | Reference | |
| Quintile 2 * age | -0.004 (-0.019; 0.012) | 0.639 | 0.000 (-0.014; 0.014) | 0.954 | 0.000 (-0.014; 0.014) | 0.984 |
| Quintile 3 * age | 0.006 (-0.009; 0.021) | 0.436 | 0.008 (-0.006; 0.022) | 0.272 | 0.008 (-0.006; 0.022) | 0.279 |
| Quintile 4 * age | 0.002 (-0.015; 0.018) | 0.846 | 0.003 (-0.011; 0.018) | 0.654 | 0.004 (-0.011; 0.019) | 0.565 |
| Quintile 5 * age | 0.025 (0.007; 0.042) | 0.005 | 0.019 (0.003; 0.035) | 0.019 | 0.020 (0.004; 0.037) | 0.016 |
| *P* for trend |  | 0.023 |  | 0.010 |  | 0.029 |

B, estimate; CI, confidence interval PHDI, Planetary Health Diet Index

Estimate, confidence intervals, and p-values were calculated using linear mixed-effects models (two-sided).

Model 1: Linear mixed-effects model adjusted for age, gender, education, marriage, and residency, region, and household income per capita.

Model 2: Linear mixed-effects model adjusted for age, s gender, education, marriage, and residency, region, household income per capita, smoking, alcohol consumption, and BMI category.

**Supplementary Table 4 Sensitivity analysis on the associations between quintiles of adherence to the cumulative average PHDI and annual cognitive decline after further adjustment for hypertension, diabetes, cardiovascular disease, daily socialization and total calories.**

|  | **Model 3** | |
| --- | --- | --- |
|  | **β (95% CI)** | ***P*** |
| **Memory** |  |  |
| Quintile 1 * age | Reference | |
| Quintile 2 * age | 0.009 (-0.012; 0.031) | 0.395 |
| Quintile 3 * age | 0.010 (-0.011; 0.031) | 0.356 |
| Quintile 4 * age | 0.026 (0.003; 0.048) | 0.027 |
| Quintile 5 * age | 0.024 (-0.001; 0.049) | 0.057 |
| *P* for trend |  | 0.058 |
| **Attention** |  |  |
| Quintile 1 * age | Reference | |
| Quintile 2 * age | 0.000 (-0.025; 0.025) | 0.991 |
| Quintile 3 * age | 0.015 (-0.010; 0.041) | 0.230 |
| Quintile 4 * age | -0.004 (-0.030; 0.023) | 0.786 |
| Quintile 5 * age | 0.011 (-0.018; 0.040) | 0.447 |
| *P* for trend |  | 0.385 |
| **Calculation** |  |  |
| Quintile 1 * age | Reference | |
| Quintile 2 * age | -0.007 (-0.028; 0.014) | 0.531 |
| Quintile 3 * age | 0.006 (-0.027; 0.015) | 0.581 |
| Quintile 4 * age | -0.012 (-0.034; 0.011) | 0.304 |
| Quintile 5 * age | 0.023 (-0.001; 0.048) | 0.064 |
| *P* for trend |  | 0.443 |
| **Cognition** |  |  |
| Quintile 1 * age | Reference | |
| Quintile 2 * age | -0.001 (-0.015; 0.014) | 0.939 |
| Quintile 3 * age | 0.006 (-0.008; 0.020) | 0.404 |
| Quintile 4 * age | 0.003 (-0.012; 0.018) | 0.714 |
| Quintile 5 * age | 0.020 (0.003; 0.036) | 0.019 |
| *P* for trend |  | 0.067 |

B, estimate; CI, confidence interval PHDI, Planetary Health Diet Index

Estimate, confidence intervals, and p-values were calculated using linear mixed-effects models (two-sided).

Model 3: Linear mixed-effects model adjusted for age, s gender, education, marriage, and residency, region, household income per capita, smoking, alcohol consumption, BMI category, hypertension, diabetes, cardiovascular disease, daily socialization and total calories.

**Supplementary Table 5 Sensitivity analysis on the associations between quintiles of adherence to the cumulative average PHDI and annual cognitive decline using time from waves as timescale.**

|  | **Unadjusted** | | **Model 1** | | **Model 2** | |
| --- | --- | --- | --- | --- | --- | --- |
|  | **β (95% CI)** | ***P*** | **β (95% CI)** | ***P*** | **β (95% CI)** | ***P*** |
| **Memory** |  |  |  |  |  |  |
| Quintile 1 * wave | Reference | | Reference | | Reference | |
| Quintile 2 * wave | 0.025 (-0.045; 0.094) | 0.485 | 0.028 (-0.038; 0.094) | 0.403 | 0.028 (-0.038; 0.094) | 0.404 |
| Quintile 3 * wave | 0.027 (-0.042; 0.097) | 0.441 | 0.027 (-0.039; 0.094) | 0.423 | 0.030 (-0.036; 0.097  ) | 0.371 |
| Quintile 4 * wave | 0.067 (-0.006; 0.140) | 0.073 | 0.074 (0.005; 0.144) | 0.036 | 0.074 (0.005; 0.143) | 0.037 |
| Quintile 5 * wave | 0.099 (0.021; 0.177) | 0.013 | 0.082 (0.008; 0.156) | 0.031 | 0.080 (0.006; 0.154) | 0.035 |
| **Attention** |  | | | | | |
| Quintile 1 * wave | Reference | | Reference | | Reference | |
| Quintile 2 * wave | -0.013 (-0.090; 0.064) | 0.743 | -0.006 (-0.082; 0.070) | 0.876 | -0.004 (-0.081; 0.072) | 0.909 |
| Quintile 3 * wave | 0.034 (-0.043; 0.112) | 0.385 | 0.039 (-0.039; 0.115) | 0.331 | 0.040 (-0.037; 0.117) | 0.305 |
| Quintile 4 * wave | -0.031 (-0.111; 0.050) | 0.455 | -0.016 (-0.096; 0.063) | 0.687 | -0.014 (-0.094; 0.065) | 0.727 |
| Quintile 5 * wave | 0.042 (-0.044; 0.129) | 0.338 | 0.031 (-0.055; 0.115) | 0.488 | 0.031 (-0.054; 0.116) | 0.477 |
| **Calculation** |  | | | | | |
| Quintile 1 * wave | Reference | | Reference | | Reference | |
| Quintile 2 * wave | -0.021 (-0.089; 0.047) | 0.546 | -0.018 (-0.084; 0.048) | 0.599 | -0.018 (-0.084; 0.049) | 0.602 |
| Quintile 3 * wave | 0.014 (-0.082; 0.055) | 0.692 | 0.011 (-0.078; 0.055) | 0.740 | 0.010 (-0.077; 0.056) | 0.764 |
| Quintile 4 * wave | -0.034 (-0.037; 0.010) | 0.358 | -0.025 (-0.094; 0.045) | 0.488 | -0.025 (-0.094; 0.045) | 0.486 |
| Quintile 5 * wave | 0.086 (0.009; 0.163) | 0.028 | 0.070 (-0.004; 0.145) | 0.064 | 0.024 (-0.004; 0.145) | 0.063 |
| **Cognition** |  | | | | | |
| Quintile 1 * wave | Reference | | Reference | | Reference | |
| Quintile 2 * wave | -0.009 (-0.056; 0.038) | 0.706 | -0.002 (-0.045; 0.042) | 0.946 | -0.001 (-0.044; 0.043) | 0.970 |
| Quintile 3 * wave | 0.019 (-0.028; 0.065) | 0.438 | 0.019 (-0.025; 0.063) | 0.394 | 0.021 (-0.023; 0.065) | 0.344 |
| Quintile 4 * wave | -0.004 (-0.052; 0.045) | 0.884 | 0.007 (-0.038; 0.053) | 0.753 | 0.008 (-0.038; 0.054) | 0.726 |
| Quintile 5 * wave | 0.075 (0.023; 0.127) | 0.005 | 0.062 (0.013; 0.111) | 0.013 | 0.062 (0.004; 0.037) | 0.013 |

B, estimate; CI, confidence interval PHDI, Planetary Health Diet Index

Estimate, confidence intervals, and p-values were calculated using linear mixed-effects models (two-sided).

Model 1: Linear mixed-effects model adjusted for age, gender, education, marriage, and residency, region, and household income per capita.

Model 2: Linear mixed-effects model adjusted for age, s gender, education, marriage, and residency, region, household income per capita, smoking, alcohol consumption, and BMI category.

**Supplementary Table 6 Sensitivity analysis on the associations between quintiles of adherence to the cumulative average PHDI* at the study baseline and annual cognitive decline.**

|  | **Unadjusted** | | **Model 1** | | **Model 2** | |
| --- | --- | --- | --- | --- | --- | --- |
|  | **β (95% CI)** | ***P*** | **β (95% CI)** | ***P*** | **β (95% CI)** | ***P*** |
| **Memory** |  |  |  |  |  |  |
| Quintile 1 * age | Reference | | Reference | | Reference | |
| Quintile 2 * age | 0.024 (-0.001; 0.048) | 0.058 | 0.024 (0.000; 0.048) | 0.047 | 0.025 (0.002; 0.048) | 0.036 |
| Quintile 3 * age | -0.007 (-0.030; 0.017) | 0.563 | -0.008 (-0.030; 0.015) | 0.511 | -0.007 (-0.029; 0.016) | 0.559 |
| Quintile 4 * age | -0.023 (-0.046; 0.001) | 0.057 | -0.016 (-0.039; 0.007) | 0.176 | -0.016 (-0.039; 0.007) | 0.164 |
| Quintile 5 * age | 0.005 (-0.019; 0.029) | 0.698 | 0.003 (-0.020; 0.027) | 0.768 | 0.004 (-0.019; 0.027) | 0.719 |
| **Attention** |  | | | | | |
| Quintile 1 * age | Reference | | Reference | | Reference | |
| Quintile 2 * age | 0.012 (-0.017; 0.040) | 0.412 | 0.011 (-0.016; 0.039) | 0.424 | 0.012 (-0.016; 0.040) | 0.400 |
| Quintile 3 * age | 0.002 (-0.025; 0.029) | 0.893 | 0.000 (-0.026; 0.027) | 0.985 | 0.001 (-0.026; 0.027) | 0.956 |
| Quintile 4 * age | -0.025 (-0.053; 0.002) | 0.070 | -0.022 (-0.049; 0.005) | 0.106 | -0.022 (-0.049; 0.005) | 0.104 |
| Quintile 5 * age | -0.028 (-0.056; 0.000) | 0.052 | -0.030 (-0.057; -0.002) | 0.033 | -0.030 (-0.057; -0.003) | 0.032 |
| **Calculation** |  | | | | | |
| Quintile 1 * age | Reference | | Reference | | Reference | |
| Quintile 2 * age | 0.008 (-0.016; 0.032) | 0.508 | 0.011 (-0.013; 0.035) | 0.361 | 0.011 (-0.012; 0.035) | 0.353 |
| Quintile 3 * age | -0.006 (-0.029; 0.017) | 0.606 | -0.005 (-0.028; 0.017) | 0.654 | -0.005 (-0.028; 0.017) | 0.655 |
| Quintile 4 * age | 0.002 (-0.021; 0.026) | 0.847 | 0.007 (-0.015; 0.030) | 0.530 | 0.007 (-0.016; 0.030) | 0.536 |
| Quintile 5 * age | 0.014 (-0.010; 0.037) | 0.262 | 0.013 (-0.010; 0.036) | 0.267 | 0.014 (-0.009; 0.037) | 0.247 |
| **Cognition** |  | | | | | |
| Quintile 1 * age | Reference | | Reference | | Reference | |
| Quintile 2 * age | 0.014 (-0.002; 0.031) | 0.090 | 0.015 (-0.001; 0.031) | 0.059 | 0.016 (0.000; 0.032) | 0.049 |
| Quintile 3 * age | -0.008 (-0.024; 0.008) | 0.331 | -0.006 (-0.021; 0.009) | 0.446 | -0.006 (-0.021; 0.010) | 0.474 |
| Quintile 4 * age | -0.018 (-0.034; -0.002) | 0.029 | -0.011 (-0.026; 0.004) | 0.147 | -0.011 (-0.027; 0.004) | 0.138 |
| Quintile 5 * age | -0.008 (-0.024; 0.009) | 0.346 | -0.006 (-0.021; 0.010) | 0.459 | -0.006 (-0.021; 0.010) | 0.475 |

B, estimate; CI, confidence interval PHDI, Planetary Health Diet Index

Estimate, confidence intervals, and p-values were calculated using linear mixed-effects models (two-sided).

*****PHDI score ranged from 0 to 14 and was categorized into quintiles.

Model 1: Linear mixed-effects model adjusted for age, gender, education, marriage, and residency, region, and household income per capita.

Model 2: Linear mixed-effects model adjusted for age, s gender, education, marriage, and residency, region, household income per capita, smoking, alcohol consumption, and BMI category.

**Supplementary Table 7 Sensitivity analysis on the associations between quintiles of adherence to the cumulative average PHDI and annual cognitive decline by using the dataset with imputation.**

|  | **Unadjusted** | | **Model 1** | | **Model 2** | |
| --- | --- | --- | --- | --- | --- | --- |
|  | **β (95% CI)** | ***P*** | **β (95% CI)** | ***P*** | **β (95% CI)** | ***P*** |
| **Memory** |  |  |  |  |  |  |
| Quintile 1 * age | Reference | | Reference | | Reference | |
| Quintile 2 * age | -0.009 (-0.083; 0.065) | 0.813 | -0.013 (-0.082; 0.057) | 0.721 | -0.013 (-0.082; 0.056) | 0.712 |
| Quintile 3 * age | -0.054 (-0.131; 0.022) | 0.163 | -0.055 (-0.127; 0.017) | 0.133 | -0.060 (-0.131; 0.011) | 0.139 |
| Quintile 4 * age | 0.079 (-0.008; 0.149) | 0.029 | 0.050 (-0.017; 0.117) | 0.144 | 0.050 (-0.016; 0.117) | 0.100 |
| Quintile 5 * age | 0.115 (0.043; 0.186) | 0.002 | 0.087 (0.020; 0.155) | 0.011 | 0.086 (0.019; 0.152) | 0.012 |
| **Attention** |  | | | | | |
| Quintile 1 * age | Reference | | Reference | | Reference | |
| Quintile 2 * age | 0.001 (-0.023; 0.026) | 0.913 | 0.000 (-0.024; 0.024) | 0.987 | 0.000 (-0.024; 0.024) | 0.981 |
| Quintile 3 * age | 0.003 (-0.022; 0.027) | 0.831 | 0.003 (-0.022; 0.027) | 0.831 | 0.003 (-0.021; 0.027) | 0.795 |
| Quintile 4 * age | -0.004 (-0.031; 0.023) | 0.769 | -0.002 (-0.028; 0.024) | 0.893 | -0.001 (-0.028; 0.025) | 0.914 |
| Quintile 5 * age | 0.003 (-0.026; 0.032) | 0.839 | 0.002 (-0.026; 0.031) | 0.878 | 0.002 (-0.026; 0.031) | 0.868 |
| **Calculation** |  | | | | | |
| Quintile 1 * age | Reference | | Reference | | Reference | |
| Quintile 2 * age | 0.032 (-0.035; 0.099) | 0.347 | 0.018 (-0.047; 0.083) | 0.579 | 0.019 (-0.046; 0.084) | 0.558 |
| Quintile 3 * age | 0.002 (-0.065; 0.069) | 0.961 | -0.010 (-0.076; 0.055) | 0.755 | -0.009 (-0.074; 0.056) | 0.783 |
| Quintile 4 * age | 0.027 (-0.043; 0.096) | 0.447 | 0.018 (-0.050; 0.085) | 0.609 | 0.018 (-0.049; 0.086) | 0.599 |
| Quintile 5 * age | 0.093 (0.021; 0.164) | 0.011 | 0.081 (0.011; 0.151) | 0.023 | 0.080 (0.011; 0.150) | 0.024 |
| **Cognition** |  | | | | | |
| Quintile 1 * age | Reference | | Reference | | Reference | |
| Quintile 2 * age | 0.009 (-0.042; 0.061) | 0.716 | 0.006 (-0.041; 0.053) | 0.807 | 0.004 (-0.043; 0.052) | 0.860 |
| Quintile 3 * age | 0.010 (-0.040; 0.060) | 0.696 | 0.004 (-0.042; 0.050) | 0.863 | 0.005 (-0.041; 0.051) | 0.831 |
| Quintile 4 * age | 0.076 (0.028; 0.123) | 0.002 | 0.058 (0.013; 0.102) | 0.011 | 0.058 (0.013; 0.102) | 0.011 |
| Quintile 5 * age | 0.064 (0.016; 0.111) | 0.008 | 0.044 (-0.001; 0.088) | 0.053 | 0.045 (0.000; 0.089) | 0.048 |

B, estimate; CI, confidence interval PHDI, Planetary Health Diet Index

Estimate, confidence intervals, and p-values were calculated using linear mixed-effects models (two-sided).

Model 1: Linear mixed-effects model adjusted for age, gender, education, marriage, and residency, region, and household income per capita.

Model 2: Linear mixed-effects model adjusted for age, s gender, education, marriage, and residency, region, household income per capita, smoking, alcohol consumption, and BMI category.

**Supplementary Table 8 Sensitivity analysis on the associations between quintiles of adherence to the cumulative average PHDI * at the study baseline and annual cognitive decline stratified according to physical activity.**

|  | **Light (n=1818)** | | **Moderate (n=677)** | | **Vigorous (n=598)** | |
| --- | --- | --- | --- | --- | --- | --- |
|  | **β (95% CI)** | ***P*** | **β (95% CI)** | ***P*** | **β (95% CI)** | ***P*** |
| **Memory** |  |  |  |  |  |  |
| Quintile 1 * age | Reference | | Reference | | Reference | |
| Quintile 2 * age | 0.018 (-0.016; 0.052) | 0.299 | 0.036 (-0.015; 0.086) | 0.170 | 0.030 (-0.025; 0.086) | 0.288 |
| Quintile 3 * age | 0.000 (-0.032; 0.033) | 0.976 | 0.007 (-0.041; 0.054) | 0.781 | 0.016 (-0.078; 0.049) | 0.658 |
| Quintile 4 * age | 0.034 (0.003; 0.066) | 0.034 | 0.011 (-0.036; 0.058) | 0.649 | 0.058 (0.005; 0.111) | 0.033 |
| Quintile 5 * age | -0.002 (-0.041; 0.037) | 0.923 | 0.027 (-0.028; 0.082) | 0.335 | 0.000 (-0.059; 0.059) | 0.997 |
| *P* for trend |  | 0.392 |  | 0.651 |  | 0.121 |
| **Attention** |  | | | | | |
| Quintile 1 * age | Reference | | Reference | | Reference | |
| Quintile 2 * age | 0.015 (-0.021; 0.051) | 0.417 | -0.027 (-0.085; 0.031) | 0.364 | 0.050 (-0.017; 0.117) | 0.145 |
| Quintile 3 * age | 0.005 (-0.030; 0.040) | 0.781 | -0.019 (-0.073; 0.035) | 0.486 | 0.021 (-0.043; 0.085) | 0.515 |
| Quintile 4 * age | -0.021 (-0.054; 0.013) | 0.236 | 0.060 (0.007; 0.114) | 0.028 | 0.057 (-0.019; 0.133) | 0.142 |
| Quintile 5 * age | -0.008 (-0.049; 0.033) | 0.692 | 0.070 (0.007; 0.133) | 0.029 | 0.021 (-0.049; 0.092) | 0.557 |
| *P* for trend |  | 0.245 |  | 0.024 |  | 0.184 |
| **Calculation** |  | | | | | |
| Quintile 1 * age | Reference | | Reference | | Reference | |
| Quintile 2 * age | 0.008 (-0.023; 0.039) | 0.607 | 0.031 (-0.019; 0.081) | 0.221 | 0.011 (-0.050; 0.072) | 0.717 |
| Quintile 3 * age | -0.005 (-0.035; 0.024) | 0.724 | -0.008 (-0.055; 0.038) | 0.731 | 0.003 (-0.055; 0.060) | 0.932 |
| Quintile 4 * age | 0.006 (-0.023; 0.035) | 0.685 | 0.018 (-0.028; 0.064) | 0.436 | -0.005 (-0.074; 0.064) | 0.891 |
| Quintile 5 * age | 0.012 (-0.023; 0.048) | 0.499 | 0.007 (-0.047; 0.061) | 0.803 | 0.036 (-0.028; 0.101) | 0.265 |
| *P* for trend |  | 0.576 |  | 0.801 |  | 0.769 |
| **Cognition** |  | | | | | |
| Quintile 1 * age | Reference | | Reference | | Reference | |
| Quintile 2 * age | 0.011 (-0.008; 0.031) | 0.260 | -0.025 (-0.057; 0.007) | 0.121 | 0.031 (-0.008; 0.069) | 0.115 |
| Quintile 3 * age | 0.018 (-0.002; 0.037) | 0.077 | -0.028 (-0.059; 0.004) | 0.085 | -0.012 (-0.048; 0.025) | 0.533 |
| Quintile 4 * age | 0.016 (-0.005; 0.036) | 0.127 | -0.016 (-0.049; 0.017) | 0.343 | 0.013 (-0.031; 0.056) | 0.573 |
| Quintile 5 * age | 0.028 (0.005; 0.051) | 0.016 | -0.011 (-0.046; 0.024) | 0.525 | 0.020 (-0.021; 0.060) | 0.337 |
| *P* for trend |  | 0.044 |  | 0.502 |  | 0.220 |

B, estimate; CI, confidence interval PHDI, Planetary Health Diet Index

Estimate, confidence intervals, and p-values were calculated using linear mixed-effects models (two-sided).

*****PHDI score ranged from 0 to 14 and was categorized into quintiles.

Linear mixed-effects model adjusted for age, s gender, education, marriage, and residency, region, household income per capita, smoking, alcohol consumption, and BMI category.

*P* for trend using the Wald test.
